# Isolation and Characterization of Articular Cartilage-Derived Cells Obtained by Arthroscopic Cartilage Biopsy from Non-Osteoarthritic Patients

**DOI:** 10.3390/cells14110830

**Published:** 2025-06-03

**Authors:** Pedro Nogueira Giglio, Débora Levy, Phelipe Oliveira Favaron, Lucas da Ponte Melo, Cadiele Oliana Reichert, Fábio Alessandro de Freitas, Juliana Sampaio Silva, Walcy Paganelli Rosolia Teodoro, Sérgio Paulo Bydlowski, Marco Kawamura Demange

**Affiliations:** 1 Instituto de Ortopedia e Traumatologia, Hospital das Clinicas, Faculdade de Medicina, Universidade de Sao Paulo-HCFMUSP, Sao Paulo 05403-010, Brazil; lucasmelomed@gmail.com (L.d.P.M.); demange@usp.br (M.K.D.); 2 Lipids, Oxidation and Cell Biology Team, Laboratory of Immunology (LIM19), Heart Institute (InCor), Faculdade de Medicina, Universidade de Sao Paulo-HCFMUSP, Sao Paulo 05403-010, Brazil; d.levy@hc.fm.usp.br (D.L.); kadielli@hotmail.com (C.O.R.); fabio.alessandro@alumni.usp.br (F.A.d.F.); jukisbio@gmail.com (J.S.S.); 3 Center of Biological Sciences, Departament of General Biology, Universidade Estadual de Londrina, Paraná 86057-970, Brazil; phelipe.favaron@yahoo.com.br; 4 Laboratory of Extracelular Matrix, Reumatology Discipline and Bioterium of the Department of Clinical Medicine, Faculdade de Medicina, Universidade de Sao Paulo-HCFMUSP, Sao Paulo 01246-903, Brazil; walcy.teodoro@fm.usp.br; 5 National Institute of Science and Technology in Regenerative Medicine (INCT-Regenera), CNPq, Rio de Janeiro 21941-902, Brazil

**Keywords:** cartilage-derived cells, cartilage repair, mesenchymal stem cells, cartilage explants, enzymatic cartilage digestion

## Abstract

Cartilage-derived migratory cells show great potential for autologous use in cartilage repair surgery. However, their collection through arthroscopic biopsy has not been previously reported in individuals without osteoarthritis. This study aimed to characterize migratory cartilage cells isolated from arthroscopic biopsies of volunteers without osteoarthritis and compare them with cells obtained by enzymatic digestion. Cell cultures were successfully established using both methods—enzymatic digestion and cell migration—from cartilage explants, with no significant differences observed in stem cell markers or plasticity between the cell lines. Cells derived from both procedures exhibited characteristics of mesenchymal stem cell, including fibroblast-like morphology, expression of CD29, CD90, and CD105 markers, absence of hematopoietic and endothelial cell markers, and the ability to differentiate into adipocytes, chondrocytes, and osteoblasts under appropriate conditions. Cells obtained by migration showed lower expression of collagen I and II, along with reduce collagen II/collagen I ratio, both positively associated with chondral matrix production, as well as lower RUNX2 expression. However, no differences were found in the levels of SOX9, essential for chondrogenic differentiation, or in the expression of *perlecan* gene. *Syndecan-1* expression was lower in cells obtained by migration. In conclusion, this study demonstrates that cartilage-derived migratory cells can be successfully obtained from arthroscopic biopsies of individuals without osteoarthritis, presenting comparable dedifferentiation and plasticity profiles. Furthermore, these cells express essential chondrogenic markers and proteins. Although further in vivo studies are needed to determine their effective regenerative potential, cartilage-derived migratory cells represent a promising avenue for cartilage repair strategies.

## 1. Introduction

Focal cartilage defects pose a significant challenge in orthopedic surgery due to their high prevalence, clinically important symptoms and functional impairment, low potential for natural repair, and tendency to progress to osteoarthritis [1]. Cell therapy techniques for cartilage treatment are of great interest because of their potential for hyaline cartilage regeneration [2]. Autologous chondrocyte implantation involves enzymatically digesting a cartilage fragment obtained through arthroscopic biopsy, followed by cell culture and implantation in a second surgical procedure. It has been successfully used as a first-line therapy for treating focal chondral defects [1].

More recently, many techniques, such as cell migration capacity from a chondral explant [3], have been developed to isolate cartilage cells, aiming to select subpopulations with specific properties. These cells, often referred to as migratory chondral progenitors, are considered promising for cell therapy for chondral defects [4].

However, most studies describing the methodology of migratory cells have been performed using cartilage derived from osteoarthritic joints [3,5,6,7,8,9,10]. This contrasts with the usual clinical context for cartilage repair surgeries, as they are performed in patients with focal cartilage defects, but not osteoarthritis [1]. So far, there is only one report of isolation of these migratory cells from non-osteoarthritic joints, using deceased donors [11]. Thus, it would be important to have evidence of the feasibility of obtaining migratory cartilage cells from arthroscopic joint biopsies in patients without knee osteoarthritis.

Therefore, the aim of this study was to obtain and characterize migratory cartilage cells from arthroscopic joint biopsies of non-diseased cartilage and compared them with cartilage cells obtained from enzymatic digestion.

## 2. Materials and Methods

### 2.1. Samples

This study received approval from the Institutional Ethics Committee (No. 46438821.2.0000.0068) and was conducted in accordance with the principles outlined in the Declaration of Helsinki. Informed consent was obtained from eight volunteers (five men and three women, aged 18–55 years) who underwent knee arthroscopy for ligament or meniscus injury treatment. None had a history of knee osteoarthritis, inflammatory disease, or infection.

Following arthroscopic inspection, a 5–10 mm cartilage fragment was extracted from a non-weight-bearing area on the lateral edge of the trochlea or intercondylar notch using a specialized arthroscopic gouge. Cell isolation was performed via either enzymatic digestion (collagenase group, four cartilage samples) or explant cell migration (explant group, four samples). There was no significant age difference between the groups (35.0 ± 12.0 vs. 32.0 ± 12.0 years). Data are presented as mean ± SEM from three independent experiments conducted in duplicate for each patient under each tested condition.

### 2.2. Enzymatic Digestion

Samples (collagenase group) were washed with a phosphate-buffered saline solution (PBS—Invitrogen, Waltham, MA, USA) containing antibiotics (penicillin 100 IU/mL and streptomycin 100 μg/mL—Sigma-Aldrich, St. Louis, MO, USA). Mechanical dissociation of the samples was performed and the fragments were transferred to a 15 mL conical plastic tube (Corning, New York, NY, USA) containing 5 mL of 0.3% (*w*/*v*) collagenase type IV solution (StemCell Technologies, Vancouver, BC, Canada), followed by incubation at 37 °C under agitation for 2 h. The sample was washed with PBS. The cells were grown in DMEM low glucose (Sigma-Aldrich, St. Louis, MO, USA), supplemented with penicillin 100 IU/mL, streptomycin 100 μg/mL and 20% fetal bovine serum (FBS—Vitrocell, Campinas, Brazil) was added. The cells were kept in an incubator at 37 °C, in a humid atmosphere containing 5% CO_2_. Cells were used for experiments at the 4th passage.

### 2.3. Explant Cell Migration

The explant group samples were obtained as previously described [12]. Briefly, the samples were washed in PBS, mechanically dissociated into homogeneous fragments (≤1 mm), and incubated at 37 °C for 15 min. Subsequently, 15 mL of DMEM low-glucose medium supplemented with 20% fetal bovine serum, 100 μg/mL streptomycin and 100 IU/mL penicillin was added gradually. The samples were then incubated at 37 °C for 72 h in a humid atmosphere with 5% CO_2_. Following incubation, the medium was replaced, and non-adherent cartilage fragments were removed. Cells were used for experiments at the fourth passage.

### 2.4. Cell Characterization

Cells were evaluated for their plasticity and multipotent potential by culturing in osteogenic (21 days), adipogenic (14 days), and chondrogenic (14 days) using differentiation commercial medium from Gibco (Waltham, MA, USA). Differentiation was confirmed by positive staining for Alizarin red, Oil Red O, and Safranin O, respectively [13]. Cell surface markers were analyzed using flow cytometry (FACSCanto flow cytometer, BD Biosciences, Franklin Lakes, NJ, USA). The monoclonal antibodies were CD29 (CD2004-R-PE), CD14 (MHCD1404-RPE), CD105 (MHCD10504R-PE), CD34 (CD34-581-01-FITC), CD117 (CD11704-RPE), CD45 (MHCD4504R-PE), CD80 (MHCD8001-FITC), CD90 (11-0909-42-FITC), and HLA-DR (11-9956-4-FITC), following the manufacturer’s protocol (Invitrogen, Waltham, MA, USA). Data acquisition was performed using the FACS Canto II flow cytometer (Becton Dickinson, Franklin Lakes, NJ, USA), with 10,000 events recorded per acquisition. Fluorescence intensity was analyzed using FlowJo V.10 software (Becton Dickinson, Franklin Lakes, NJ, USA) and expressed as the percentage of positively stained cells.

### 2.5. Protein Detection by Indirect Immunofluorescence

Indirect immunofluorescence was used to evaluate the expression of collagen I, collagen II, PPARγ, RUNX2, and SOX9 as previous described [13]. Cells were seeded in black 96-well flat-bottom microplates (Corning, New York, NY, USA) for 24 h. Following incubation, cells were washed with Dulbecco’s Phosphate-Buffered Saline (DPBS) and fixed with 4% paraformaldehyde (Sigma-Aldrich, St. Louis, MO, USA). Cells were permeabilized using 0.1% Triton X-100 solution (Sigma-Aldrich, St. Louis, MO, USA) followed by blocking with 5% BSA (Sigma-Aldrich, St. Louis, MO, USA). Cells were incubated for 16 h at 4 °C with the antibodies listed in Supplementary Table S1 (collagen I, collagen II, PPARγ, RUNX2, SOX9). After incubation with anti-collagen I, collagen II, and PPARγ antibodies, cells were further treated with secondary anti-rabbit AlexaFluor^®^ 488 antibody (1:500 dilution; Molecular Probes, Eugene, OR, USA) for 2 h. Cells incubated with anti-RUNX2 and SOX9 antibodies were subsequently incubated for 2 h with secondary anti-mouse R-phycoerythrin antibody (1:500 dilution; Molecular Probes, Eugene, OR, USA). All samples were incubated with 0.1 µg/mL Hoechst 33342 dye for nuclear labeling. Analysis was performed using the ImageXpress Micro High Content Screening System (Molecular Devices, San Jose, CA, USA), with nine sites per well and two wells per treatment acquired. The percentage of positively stained cells and fluorescence intensity were determined using the Cell Scoring MetaXpress software (version 5.0, Molecular Devices, San Jose, CA, USA).

### 2.6. Gene Expression

This work is in consonance with the MIQE guidelines: minimum information for publication of quantitative real-time PCR experiments. RNA from cultured cells were extracted by TRI Reagent (Sigma-Aldrich, St. Louis, MO, USA) as previously described [13].

### 2.7. Evaluation of Genes Involved in Cell Differentiation

Real-time PCR was performed using the 7500 Fast Real-Time PCR System thermocycler (Life Technologies, Carlsbad, CA, USA). Primers were obtained from Integrated DNA Technologies (Coralville, IA, USA) as pre-designed assays for hydrolyzable probes (Supplementary Table S2). The reactions were carried out using the TaqMan Universal PCR Master Mix (Thermo Fisher, Waltham, MA, USA). Gene expression analysis related to osteogenic (*ALPL*, *RUNX2*, *osteocalcin*, *osteopontin*), adipogenic (*PPARγ* and *CEBPα*), and chondrogenic (*syndecan-1* and *perlecan*) cell differentiation was normalized to the endogenous *GAPDH* gene (Thermo Fisher, Waltham, MA, USA).

### 2.8. Data Analysis

Statistics were performed based on data obtained in tests conducted with cells isolated either by enzymatic digestion or explant. The *t*-test was performed using GraphPad Prism 8 (GradhPad Software, San Diego, CA, USA). The results were expressed as mean ± SD of two different experiments carried out in duplicate considering a statistically significant *p* value < 0.05.

## 3. Results

### 3.1. Cell Morphology

**Figure 1:**
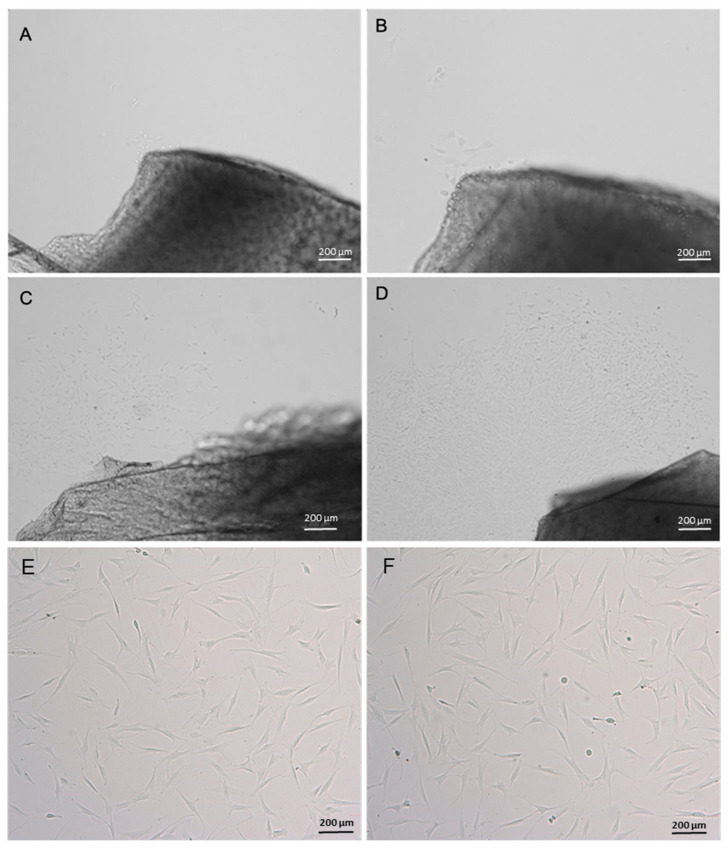
Photomicrograph of migrating cells from cartilage explant and cell culture. (**A**) Four days of culture; (**B**) nine days of culture; (**C**) twelve days of culture; (**D**) fifteen days of culture; (**E**) cell culture of explant group and (**F**) cell culture of collagenase group. Images obtained with an invert microscope Axio A.1 (Carl Zeiss, Oberkochen, Germany). Scale bar, 200 µm.

Cells were isolated and cultures were established with both enzymatic and explant migration methods. In the explant group, progressive cell migration from the tissue fragment to the plastic culture plate was observed (Figure 1A–D).

The morphology of the cells in culture was similar in both groups. The cells exhibited an elongated, fusiform and fibroblast-like format (Figure 1E,F). Cell growth was similar in both groups, with a doubling time of 2.55 days in the explant group and 2.98 days in the collagenase group.

### 3.2. Cell Characterization

**Figure 2:**
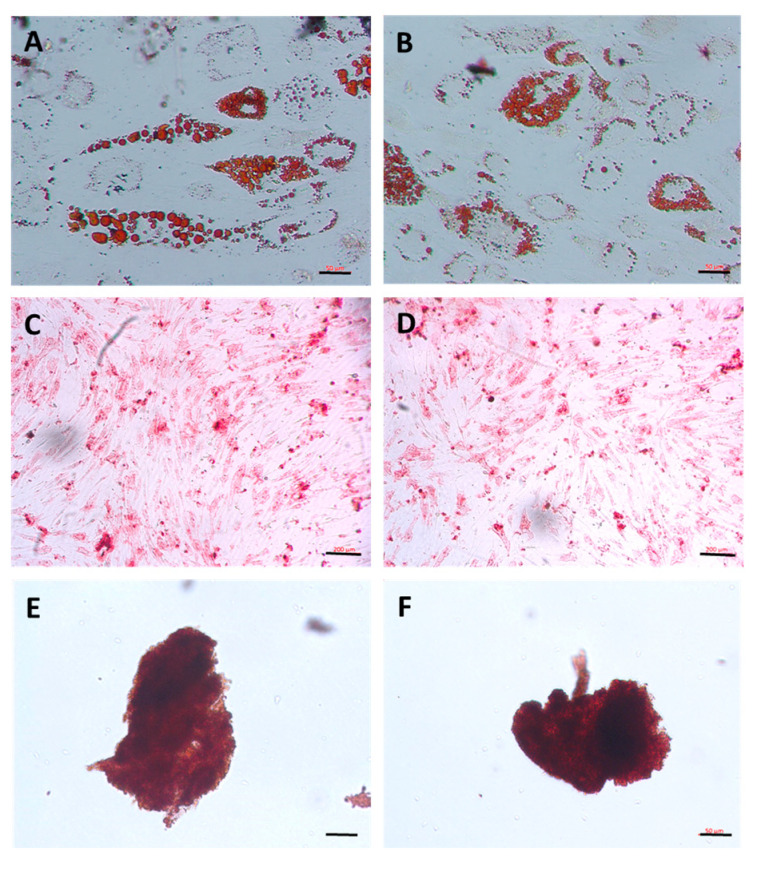
Representative images of differentiation into adipocytes, osteocytes and chondrocytes. (**A**,**B**): adipogenic differentiation (Oil Red O); (**C**,**D**): osteogenic differentiation (Alizarin Red); (**E**,**F**): chondrogenic differentiation (Safranin O). Explant group: (**A**,**C**,**E**); collagenase group: (**B**,**D**,**F**). Images obtained with inverted microscope Zeiss Axio A.1. Scale bar 50 µm in (**A**,**B**,**E**,**F**) and calibration 200 µm in (**C**,**D**).

Cells of both groups expressed the mesenchymal stem cell markers CD29, CD90 and CD105, with positivity > 95%, whereas they did not express the hematopoietic and endothelial cell markers CD14, CD34, CD45, CD80, CD117 and HLA-DR (Table 1 and Supplementary Figure S1). Moreover, cells of both groups were able to differentiate into the three mesenchymal lineages, which characterized them as multipotent cells. Adipogenic differentiation was confirmed by the lipid vacuoles stained with Oil Red (Figure 2A,B). Osteogenesis was confirmed by the deposition of fixed calcium determined by Alizarin Red staining (Figure 2C,D). Finally, chondrogenesis was confirmed by the formation of the chondrogenic cell aggregate, observed using Safranin O staining (Figure 2E,F).

### 3.3. Analysis of Proteins Analysis: Collagen I and II, RUNX2, SOX9 and PPARγ

**Figure 3:**
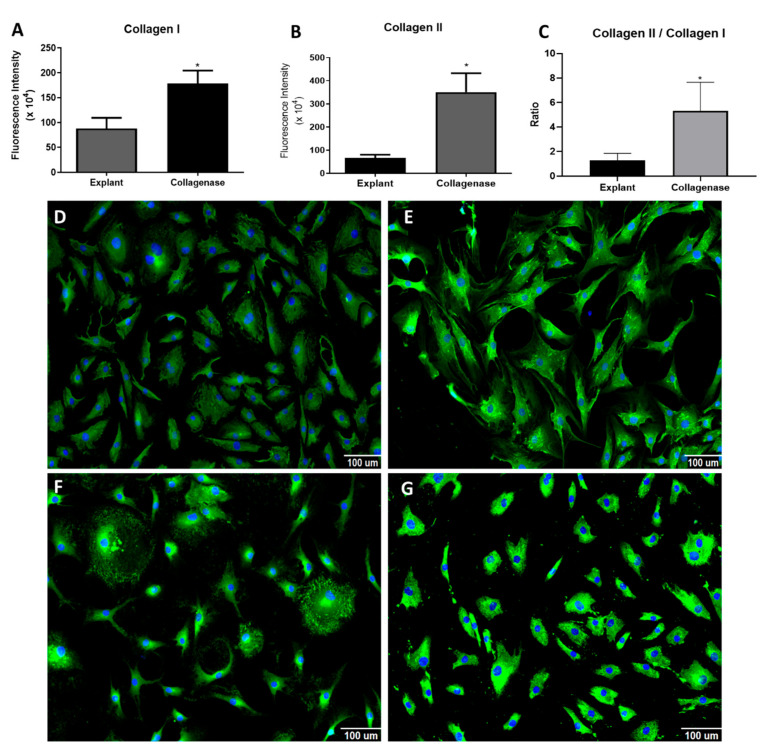
Analysis of proteins Collagen I, Collagen II. (**A**,**B**) Graph of fluorescence intensity of collagen I (**A**) and collagen II (**B**). (**C**) Graph of ratio of collagen II for collagen I. (**D**,**E**) Representative images of collagen I (green) and nucleus (blue, stained with Hoechst 33342) in explant cells (**D**) and in collagenase cells (**E**). (**F**,**G**) Representative images of collagen II (green) and nucleus (blue, stained with Hoechst 33342) in explant cells (**F**) and in collagenase cells (**G**). Statistical analysis was performed by Student’s *t*-test in the GraphPad Prism 8 software. Data are mean ± SEM from three independent experiments in duplicate. * *p* < 0.05, Scale bar, 100 µM.

Collagen I (Figure 3A,D,E) and II (Figure 3B,F,G) labeling was higher in the collagenase group than in the explant group (the collagen II/collagen I labeling ratio has been used as a marker of the chondrogenic phenotype; it was higher in the collagenase group than in the explant group (Figure 3C).

**Figure 4:**
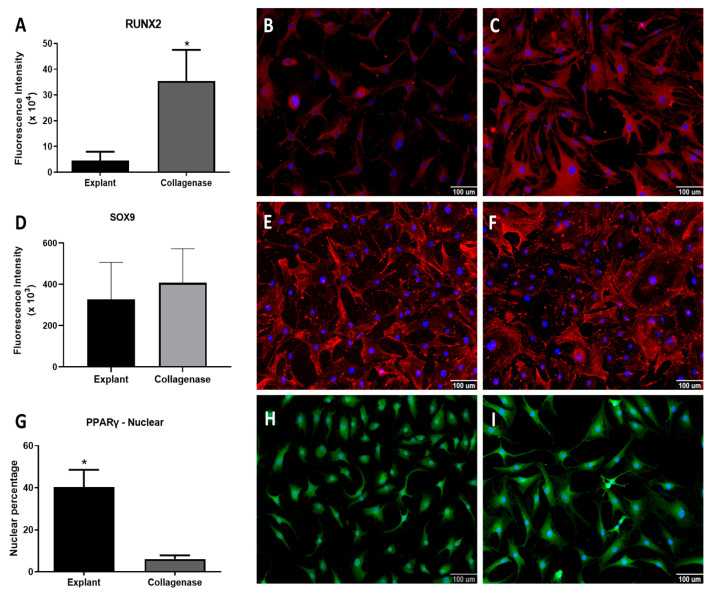
Analysis of proteins RUNX2, SOX9 and PPRAγ. (**A**) Graph of fluorescence intensity of RUNX2; (**B**,**C**) representative images of RUNX2 (red) and nucleus (blue, stained with Hoechst 33342 dye) in explant cells (**B**) and in collagenase cells (**C**). (**D**) Graph of fluorescence intensity of SOX9; (**E**,**F**) representative images of SOX9 (red) and nucleus (blue, stained with Hoechst 33342 dye) in explant cells (**E**) and in collagenase cells (**F**). (**G**) Graph of nuclear PPRAγ positivity; (**H**,**I**) representative images of PPRAγ (red) and nucleus (blue, stained with Hoechst 33342 dye) in explant cells (**H**) and in collagenase cells (**I**). Statistical analysis was performed by Student’s t-test in the GraphPad Prism 8 software. Data are mean ± SEM from three independent experiments in duplicate. * *p* < 0.05, Scale bar, 100 µM.

The transcriptional factor RUNX2 was more expressed in the collagenase group (Figure 4A–C). The transcription factor SOX9 expression, key for chondrogenic differentiation and chondral matrix production, was not different between groups (Figure 4D–F). Finally, the hormone receptor PPARγ, essential in adipogenic differentiation and inhibitor of osteogenesis, had similar marking levels in the cytoplasm of both whereas the percentage of nuclear marking was higher in the explant group (Figure 4G–I).

### 3.4. Gene Expression of Cell Differentiation Markers

The expression of genes related to the differentiation of the osteogenic, adipogenic, and chondrogenic lineages was determined with real-time PCR. RNA expression was normalized with the endogenous *GAPDH* gene expression.

**Figure 5:**
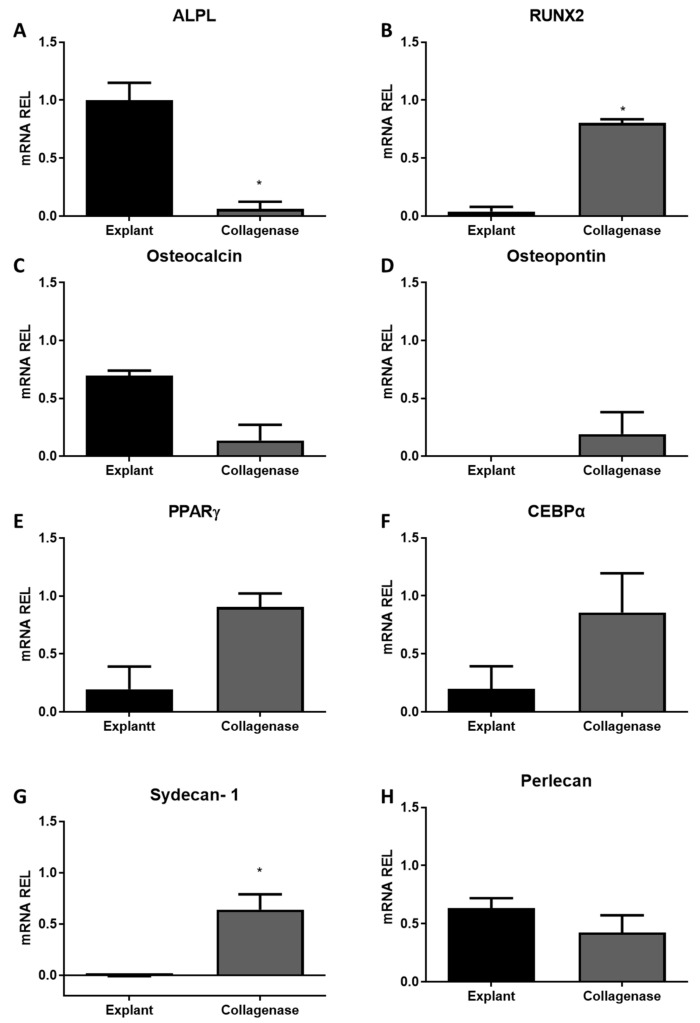
Analysis of mRNA expression for cartilage cells obtained by explantation or collagenase digestion. (**A**) *ALPL*, (**B**) *RUNX2*, (**C**) *Osteocalcin*, (**D**) *Osteopontin*; (**E**) *PPARγ*; (**F**) *CEBPα*; (**G**) *Syndecan-1* and (**H**) *Perlecan* gene. Statistical analysis was performed by Student’s *t*-test in the GraphPad Prism 8 software. Data are expressed by mean ± SEM from three independent experiments in duplicate. * *p* < 0.05.

*ALPL*, *RUNX2*, *osteocalcin*, and *osteopontin* are genes related to osteogenic differentiation. Cells of the explant group had a higher expression of the *ALPL* gene (gene for alkaline phosphatase, Figure 5A), but a lower expression of the *RUNX2* gene (Figure 5B). Expressions of *osteocalcin* and *osteopontin* were not statistically different between the groups (Figure 5C,D, respectively).

The expression of genes related to adipogenic differentiation, *PPARγ* and *CEBPα*, were not different between the groups (Figure 5E,F, respectively).

*Syndecan-1* and *perlecan* are genes related to chondrogenic differentiation. *Syndecan-1* expression was higher in the collagenase group (Figure 5G), while *perlecan* expression was similar in both groups (Figure 5H).

## 4. Discussion

**Figure 6:**
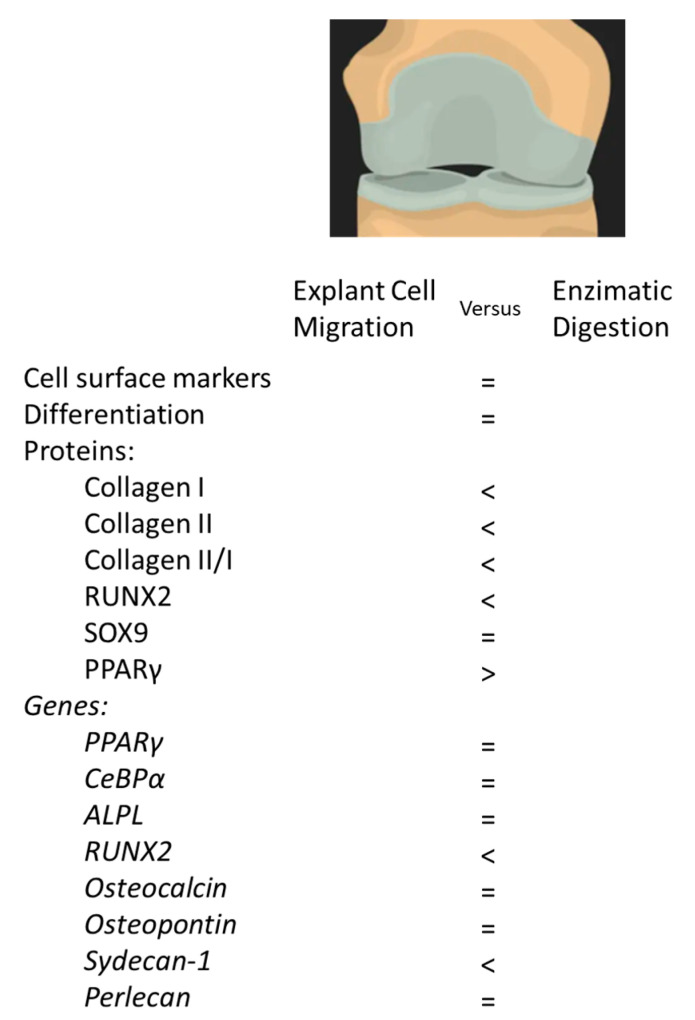
Summary of the effects of isolation methodology in cells derivate from arthroscopic joint biopsies of non-diseased cartilage. = equal expression; <: lower expression; >: higher expression.

The purpose of this study was to isolate and characterize migratory cells from cartilage explants obtained by arthroscopic biopsy of healthy, non-osteoarthritic human knees (Figure 6). These cells were compared to the current clinical standard of cartilage cells isolated by enzymatic digestion.

In the clinical context of autologous cell therapy in cartilage repair surgery, the ability to obtain cells from non-osteoarthritic cartilage is essential, as the best candidates for cartilage surgery are patients with focal chondral defects, but with no installed osteoarthritis [14].

There is controversy as to whether isolation of migratory cartilage-derived cells is feasible in joints without osteoarthritis. To date, to the best of our knowledge, this is the first study describing the establishment of cartilage cell cultures obtained by arthroscopic biopsy of non-arthritic knees.

The literature seems to point to a greater difficulty in obtaining these cells in healthy cartilage: Koelling et al. (2009) [3] described failure to obtain cells by migrating healthy cartilage explants. Wang et al. (2020) [10] demonstrated a greater number of migratory cells in areas of greater osteoarthritis injury, compared to less affected areas. Other authors have described the isolation of migratory cells from healthy bovine cartilage, but after an experimental model of trauma, which would supposedly activate or recruit these cells [15,16,17].

In the present study, cells obtained by migration or enzymatic digestion displayed a similar immunophenotype, compatible with mesenchymal stem cells (MSC). Several previous studies show similar results, describing MSC-compatible immunophenotype for migratory cartilage cells [3,11,18], and cells obtained by enzymatic digestion [19,20,21,22]. Both cartilage cells extracted by enzymatic cartilage digestion and explant migration resulted in cultured populations capable of tri-lineage differentiation (osteogenic, adipogenic and chondrogenic). These cells, therefore, can be characterized as multipotent. This has previously been reported for migratory cartilage cells [3,10,11,15], but with some controversy for enzymatically extracted cells, with some authors describing tri-lineage differentiation capacity [19,21,23,24,25,26,27], but others showing differentiation only in one or two lineages [3,20,22]. There are several potential factors that could be related to these differences: age of donors, presence of osteoarthritis, specific culture conditions, such as the medium used and time of cells in culture.

The present study analyzed the expression of factors related to osteogenic (*RUNX2, ALPL*, *osteocalcin* and *osteopontin*), chondrogenic (*SOX9*, *syndecan* and *perlecan*) and adipogenic (*PPARγ* and *CEBPα*) differentiation. RUNX2 is the main factor related to the proliferation and differentiation of osteoblasts [28]. The current study demonstrated increased expression of *RUNX2* in cells isolated by collagenase, in contrast to previous studies [3,11,15]. This difference might be related to use of cartilage from osteoarthritic joints in previous studies, since osteoarthritis is related to increased expression of this factor [29].

*SOX9* is the key factor in chondrocyte differentiation and chondral extracellular matrix production. A similar expression of SOX9 was demonstrated between the groups in the current study, which speaks in favor of a potential for differentiation and similar chondral matrix production [4,18]. Previous studies are contradictory in this regard, with some demonstrating greater expression of *SOX9* in cells obtained by enzymatic digestion than in cells obtained by explant migration [11], and others the opposite [15]. These differences may reflect specific culture conditions, such as the medium used, cell culture time and number of passages [30]. They may also be affected by tissue damage related to osteoarthritic cartilage, as the disease causes changes in the expression of this factor [31].

Finally, the factors related to adipogenic differentiation were studied: *PPARγ* and *CEBPα*. In the present study, a higher presence of PPARγ nuclear labeling was demonstrated in cells isolated by explant, but similar cytoplasmatic expression. Previously, Wanget al. (2019) [11] demonstrated higher *PPARγ* expression in chondral explant migratory cells. Acting synergistically with *PPARγ*, the *CEBPα* gene is also essential for adipogenic differentiation [32,33], and it was not different between the groups in the present study.

There was higher staining intensity on immunofluorescence for both collagen I and collagen II in cells extracted by enzymatic digestion, than in by explant migration. Additionally, the collagen II/collagen I ratio was higher in the enzymatic digestion group. The collagen II/collagen I ratio is used as a marker of the chondrogenic capacity of cells in culture [34,35]. Therefore, enzymatically extracted cells could be considered more chondrogenic. It may also indicate a less differentiated state of migratory cells [3]. Previously, other authors demonstrated more intense expression of type II collagen by cartilage cells isolated by enzymatic digestion than by explant migration [3,11,15,18].

Cartilage cells isolated by explant migration have previously been described as promising for use as a cartilage autologous cell-therapy product [4]. The current study is an essential step within the context of translation for clinical use of this technique. In it, the following key issues were investigated: (1) the feasibility of the method for isolating migratory cells and establishing cultures from arthroscopic biopsy of healthy cartilage; (2) the detailed characteristics of the cell lines obtained; (3) the direct comparison to the current standard in clinical practice for autologous cell-therapy (cartilage cells isolated by enzymatic digestion). It is important to recognize that the analysis in vitro of characteristics in cultured cells are not definitive in determining their potential in vivo in the repair of chondral lesions. Tissue regeneration depends on complex interactions between the biological environment at the site of injury and implanted cells [36,37,38].

There are some limitations in the current study. Group samples were not paired (the same donor providing a sample for each group). This would provide the advantage of reducing possible variability between the individuals in the study’s findings. However, the amount of tissue that can be safely collected in biopsies of healthy cartilage is limited, as to limit the possibility of donor site morbidity.

The characteristics of cells in cultures are not temporally stable, with important differences between fresh cells (first passage), early cultures (up to the fourth passage) and late cultures (more than five passages). Contrasting results from the literature may reflect these temporal differences, and present results could be different at other times in culture [27,34,39]. Analysis of first-pass cells is relevant because they reflect a cell condition closer to that of the original tissue. However, for the context of clinical use of cultured cells, dependent on the production of a large number of cells, more culture time is required. Thus, we evaluate all samples in the same passage (passage 4), when it is possible to obtain an adequate number of cells for cell therapy (10–20 × 10^6^ cells) for both populations.

On the other hand, this study has noteworthy strengths. We characterized cells with potential use in cell therapy for focal chondral lesions, obtained from arthroscopic biopsy of the articular cartilage of the human knee, in patients without knee osteoarthritis. Almost all the previous studies in the literature evaluating migratory cartilage cells use animal origin cells, or use material discarded from knee arthroplasties for osteoarthritis. In these works, the difference in species, diagnosis, and sample volume used may have a relevant impact on the biological characteristics of the populations obtained. To enhance the external validity of the findings for clinically relevant focal cartilage lesions, a human knee cartilage without osteoarthritis was analyzed, using small fragments collected via arthroscopic biopsy. The present study was not limited to describing and characterizing cartilage cells obtained by migration, but compared them with the current standard for clinical use, which are cells obtained by enzymatic digestion.

## 5. Conclusions

We conclude that it is feasible to isolate and establish cultures of cartilage-derived migratory cells from cartilage explant, obtained by arthroscopic biopsy of patients without osteoarthritis, as well as from enzymatic digestion of the same material. Comparative characterization between cells obtained by these two methods demonstrated a similar profile of surface markers, differentiation and plasticity. Cells extracted by enzymatic digestion had higher expression markers for chondrogenic capacity such as collagen I, collagen II/collagen I ratio and Sydecan-1.

## Figures and Tables

**Table 1:** Cell surface markers measured by flow cytometry.

	Explant	Collagenase
CD14	0.6 ± 0.06	1.2 ± 0.04
CD29	96.6 ± 0.22	95.4 ± 0.53
CD34	0.9 ± 0.10	1.3 ± 0.01
CD45	3.8 ± 0.07	2.1 ± 0.02
CD80	3.4 ± 0.05	2.9 ± 0.07
CD90	99.5 ± 0.16	98.7 ± 0.22
CD105	97.4 ± 0.36	97.8 ± 0.48
CD117	1.2 ± 0.04	0.3 ± 0.01
HLA-DR	1.8 ± 0.05	0.5 ± 0.02

Results are expressed as percentage of positive cells ± standard deviation CD29, CD90, and CD105, mesenchymal stem cell markers CD14, CD34, CD45, CD80, CD117, and HLA-Dr: hematopoietic and endothelial markers.

## Data Availability

The data generated in this research can be made available upon consultation with P.N.G., D.L. or S.P.B. The data will be made available anonymously. Data from the medical records of study participants cannot be made available.

## Supplementary Materials

The following supporting information can be downloaded at: https://www.mdpi.com/article/10.3390/cells14110830/s1, Table S1: Antibodies used in the analysis of proteins by indirect immunofluorescence; Table S2: List of primers used for real time PCR analysis; Figure S1: Representative histograms of flow cytometry data of cell surface markers.
