# Supplementary material for: Isolation and Characterization of Articular Cartilage-Derived Cells Obtained by Arthroscopic Cartilage Biopsy from Non-Osteoarthritic Patients
Source: Cells. 2025 Jun 3;14(11):830. doi: 10.3390/cells14110830 (PMC12155265; doi:10.3390/cells14110830)
Supplement: Supplementary file 1 [file cells-14-00830-s001.zip › cells-3592966-supplementary.pdf]

Table S1. Antibodies for protein analysis by indirect immunofluorescence.

Antibody	Dilution	Mark	Catalog
Collagen I	1:100	Rockland	600-401-103-0
Collagen II	1:100	Rockland	600-401-105-01
PPARγ	1:100	Novusbio	NB120-19481
RUNX2	1:100	Novusbio	H860-M06
SOX9	1:100	ABCam	Ab92494

Table S2. Primers used for real-time PCR

	Foward	Reverse	Probe
RUNX2	5'- GGCGGTCAGAGAACAAAC-3'	5'-CTTCACAAATCCTCCCCAAGT-3'	5'-/56-FAM/AACCCAGA/ZEN/CCATCAGGTTTGGGCG /31ABkFQ/-3'
ALPL	5'-TCCCTGATGTTATGCATGAGC-3'	5'-CGAGAGTTATGCATGAGC-3'	5'-/56-FAM/TGTCTGAGT/ZEN/ACCAGTCCCGGTCA /31ABkFQ/-3'
Osteocalcin	5'-CGCCTGGGTCTCTTCACT-3'	5'-CTCACACTCCTCGCCCTAT-3'	5'-/56-FAM/AGTCCAGCA/ZEN/AAGGTGCAGCCT/31ABkFQ/-3'
Osteopontin	5'-TTCAACTCCTCGCTTTCCAT-3'	5'-CCCCACAGTAGACACATATGATG-3'	5'-/56-FAM/ACCTGACAT/ZEN/CCAGTACCCTGATGCT /31ABkFQ/-3'
CEBPα	5'-CCCTCCACCTTCATGTAGAAC-3'	5'-CCACGCCTGTCCTTAGAAAG-3'	5'-/56-FAM/TCCCAAGCCC/ZEN/CAAGTCCCTATGTTTC /31ABkFQ/-3'
PPARg	5'-GGATTCAGCTGGTCGATATCAC-3'	5'-GTTTCAGAAATGCCTTGCAGT-3'	5'-/56-FAM/CTCATAATG/ZEN/GGCACAGACAGAAGC/31ABkFQ/-3'
Syndecan-1	5'-GTCTGCTGTGACAAGGTGAT-3'	5'-CTACTACTTTGCCCCTGAAGA-3'	5'-/56-FAM/TCAGAGTCA/ZEN/TCCCCAGAGCCATCT/31ABkFQ/-3'
Perlecan	5'-CGACTAGCTGGAACTCGTG-3'	5'-TTGACCAACCCGATGACTTC-3'	5'-/56-FAM/TGTGAATGT/ZEN/GACAATGCCTGCGC/31ABkFQ/-3'

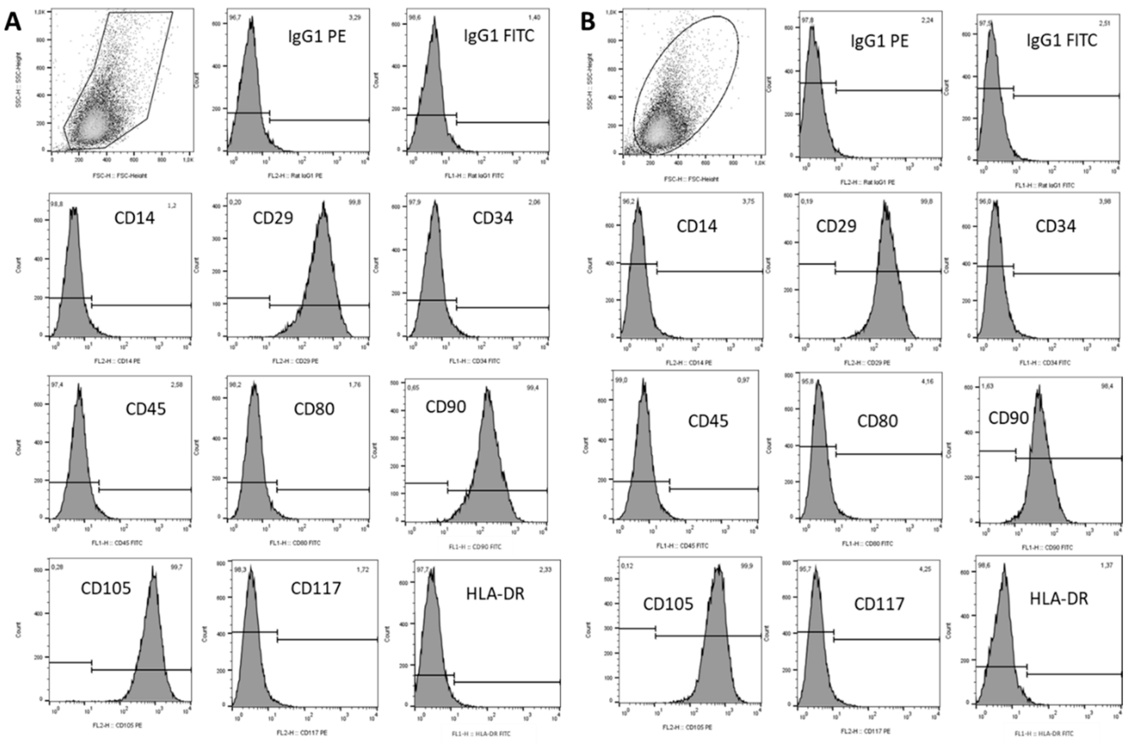

Figure S1- Representative histograms of flow cytometry data of cell surface markers. CD29, CD90, and CD105: mesenchymal stem cell markers CD14, CD34, CD45, CD80, CD117, and HLA-DR: hematopoietic and endothelial markers A: Explant cells; B: Collagenase cells.
